# Supplementary material for: The distribution of antibiotic use and its association with antibiotic resistance
Source: eLife. 2018 Dec 18;7:e39435. doi: 10.7554/eLife.39435 (PMC6307856; doi:10.7554/eLife.39435)
Supplement: Supplementary file 1. [file elife-39435-supp1.docx]

**Supplementary File for *The distribution of antibiotic use and its association with antibiotic resistance***

Scott W. Olesen, Michael L. Barnett, Derek MacFadden, Marc Lipsitch, Yonatan H. Grad

**Tables**

Table 1: Antibiotic groups

Table 2: Correlations between total use and resistance

Table 3: Systematic differences in use-resistance correlations

Table 4: Correlations between first use and repeat use

Table 5: Sensitivity of multiple regression coefficients

Table 6: Generic antibiotic formulations

Table 7: Oral and injected drug forms

**Table 1: Antibiotic groups.** MarketScan pharmacy claims were grouped according to the second column; data from antibiotic susceptibility reports were grouped according to the third.

| **Drug group** | **Use drugs** | **Resistance drugs** |
| --- | --- | --- |
| β-lactam | amoxicillin, amoxicillin/clavulanate, ampicillin, ampicillin/sulbactam, clarithromycin/amoxicillin, dicloxacillin, nafcillin, oxacillin, penicillin G, penicillin V, piperacillin/tazobactam, ticarcillin/clavulanate | amoxicillin/clavulanate, ampicillin, ampicillin/sulbactam, cloxacillin, nafcillin, piperacillin/tazobactam, ticarcillin/clavulanate, penicillin |
| cephalosporin | cefaclor, cefadroxil, cefazolin, cefdinir, cefditoren, cefepime, cefixime, cefotaxime, cefotetan, cefoxitin, cefpodoxime, cefprozil, ceftaroline, ceftazidime, ceftibuten, ceftriaxone, cefuroxime, cephalexin | cefazolin, cefepime, ceftazidime, ceftriaxone, cefuroxime, cephalexin |
| macrolide | azithromycin, clarithromycin, clarithromycin/amoxicillin, clindamycin, erythromycin, erythromycin/sulfisoxazole, fidaxomicin, lincomycin, telithromycin | azithromycin, erythromycin |
| nitrofurantoin | nitrofurantoin | nitrofurantoin |
| quinolone | ciprofloxacin, gatifloxacin, gemifloxacin, levofloxacin, moxifloxacin, nalidixic acid, norfloxacin, ofloxacin | ciprofloxacin, levofloxacin, moxifloxacin |
| tetracycline | demeclocycline, doxycycline, metronidazole/tetracycline, minocycline, tetracycline | tetracycline |
| TMP/SMX | erythromycin/sulfisoxazole, sulfadiazine, sulfisoxazole, trimethoprim, TMP/SMX | TMP/SMX |

TMP/SMX: trimethoprim/sulfamethoxazole.

**Table 2: Correlations between total use and resistance.** Main data: MarketScan data as described in the main text. Children: MarketScan data with members at most 15 years old. HRR: Medicare use data and resistance data aggregated by hospital referral region.

|  |  | **Correlation between aggregate use and resistance (Spearman’s *ρ*, %; 95% CI)** | | | | | | |
| --- | --- | --- | --- | --- | --- | --- | --- | --- |
| **Pathogen** | **Antibiotic** | **Main data** | **Days supply** | **Children** | **Older adults** | **4-year** | **Not IP only** | **OP/ER only** |
| S. pneumoniae | macrolide | 64 (41, 80) | 65 (41, 80) | 77 (59, 87) | 47 (18, 69) | 62 (37, 78) | 64 (40, 80) | 69 (20, 91) |
| E. coli | quinolone | 64 (42, 79) | 61 (39, 77) | 45 (18, 66) | 69 (49, 82) | 60 (37, 76) | 68 (48, 81) | 28 (-17, 63) |
| S. aureus | macrolide | 56 (31, 74) | 55 (29, 73) | 50 (22, 70) | 51 (24, 70) | 56 (30, 74) | 57 (31, 75) | 68 (31, 87) |
| E. aerogenes | quinolone | 54 (28, 73) | 57 (31, 75) | 35 (3.9, 59) | 53 (26, 72) | 48 (20, 69) | 54 (27, 73) | 11 (-39, 56) |
| K. pneumoniae | quinolone | 51 (25, 70) | 51 (25, 70) | 46 (19, 67) | 48 (21, 68) | 48 (21, 68) | 52 (26, 71) | 12 (-33, 52) |
| E. cloacae | quinolone | 50 (23, 70) | 53 (27, 72) | 41 (12, 63) | 50 (23, 70) | 46 (18, 67) | 52 (26, 71) | 7.1 (-41, 52) |
| E. coli | TMP/SMX | 49 (22, 68) | 41 (13, 63) | 48 (21, 68) | 36 (7.1, 59) | 41 (13, 63) | 46 (19, 67) | 32 (-12, 66) |
| A. baumannii | quinolone | 47 (18, 69) | 50 (21, 70) | 43 (13, 66) | 52 (25, 72) | 44 (14, 67) | 57 (30, 76) | -3.8 (-58, 52) |
| E. cloacae | beta-lactam | 42 (13, 64) | 36 (5.4, 60) | 32 (1.5, 57) | 31 (0.059, 56) | 37 (7.6, 61) | 41 (12, 64) | 66 (24, 87) |
| C. freundii | cephalosporin | 41 (10, 64) | 36 (5.1, 61) | 40 (9.5, 63) | 38 (6.9, 62) | 40 (9.2, 63) | 38 (7.6, 62) | 23 (-34, 68) |
| A. baumannii | cephalosporin | 39 (8, 64) | 35 (2.9, 61) | 48 (18, 69) | 24 (-9.1, 52) | 42 (11, 66) | 42 (11, 66) | 13 (-43, 62) |
| Serratia | cephalosporin | 38 (7.7, 61) | 34 (3.7, 59) | 39 (9, 62) | 29 (-1.9, 55) | 36 (5.7, 60) | 38 (7.8, 61) | 57 (11, 83) |
| E. coli | cephalosporin | 37 (8.4, 60) | 33 (4.1, 57) | 47 (20, 67) | 25 (-5.3, 51) | 33 (3.8, 57) | 38 (8.6, 61) | 22 (-24, 59) |
| E. aerogenes | beta-lactam | 37 (5.8, 62) | 28 (-4.6, 55) | 25 (-7.4, 53) | 31 (-0.54, 58) | 32 (0.48, 58) | 38 (7, 63) | 63 (18, 86) |
| A. baumannii | beta-lactam | 37 (3.3, 63) | 34 (0.72, 61) | 32 (-2.5, 59) | 18 (-17, 49) | 34 (0.17, 61) | 34 (-0.2, 61) | 22 (-40, 71) |
| C. freundii | quinolone | 36 (5.5, 60) | 41 (12, 64) | 17 (-15, 46) | 35 (3.8, 59) | 33 (1.8, 58) | 38 (7.2, 62) | 3.2 (-49, 54) |
| P. aeruginosa | quinolone | 36 (6.8, 59) | 36 (7.6, 60) | 27 (-2.7, 53) | 32 (3.1, 57) | 34 (5.3, 58) | 32 (2.4, 57) | -8.4 (-51, 37) |
| CoNS | cephalosporin | 35 (1.3, 61) | 28 (-6.2, 57) | 53 (23, 74) | 17 (-18, 48) | 37 (3.8, 63) | 40 (6.9, 66) | 8.2 (-54, 65) |
| E. aerogenes | cephalosporin | 33 (1.8, 59) | 28 (-4, 55) | 38 (7.2, 62) | 24 (-7.8, 52) | 36 (5.4, 61) | 36 (5, 61) | 41 (-10, 76) |
| S. pneumoniae | TMP/SMX | 32 (0.48, 58) | 31 (-1.1, 57) | 38 (6.5, 62) | 38 (7.4, 63) | 33 (0.97, 59) | 25 (-7.1, 53) | 77 (32, 94) |
| E. coli | beta-lactam | 31 (1.7, 56) | 24 (-6.3, 50) | 33 (3.4, 57) | 30 (0.087, 55) | 22 (-8, 49) | 27 (-2.8, 53) | -8.4 (-50, 36) |
| CoNS | macrolide | 31 (-0.63, 56) | 29 (-2.2, 55) | 27 (-4.2, 54) | 38 (7.3, 62) | 23 (-8.8, 51) | 33 (1.5, 58) | 12 (-41, 60) |
| C. freundii | beta-lactam | 31 (-2.1, 57) | 22 (-11, 51) | 19 (-15, 48) | 26 (-7.4, 54) | 27 (-6.4, 54) | 28 (-5.3, 55) | 52 (-4.8, 83) |
| E. cloacae | cephalosporin | 29 (-1.9, 54) | 23 (-7.9, 50) | 35 (5.4, 59) | 17 (-14, 45) | 29 (-1.2, 55) | 29 (-1.2, 55) | 58 (15, 82) |
| E. faecium | beta-lactam | 28 (-4.3, 55) | 22 (-10, 51) | 37 (5.4, 61) | 20 (-13, 49) | 28 (-3.9, 55) | 22 (-11, 51) | -26 (-75, 40) |
| K. oxytoca | cephalosporin | 27 (-5.3, 54) | 26 (-6.6, 53) | 31 (-0.57, 58) | 26 (-6.6, 53) | 25 (-7.9, 53) | 33 (0.42, 60) | 57 (8.4, 84) |
| CoNS | TMP/SMX | 27 (-4.4, 53) | 24 (-7.5, 51) | 27 (-4, 53) | 16 (-16, 44) | 25 (-6.7, 51) | 31 (-0.19, 57) | -3.2 (-54, 49) |
| E. faecalis | quinolone | 26 (-7.6, 55) | 27 (-7.3, 55) | 22 (-12, 51) | 40 (8.1, 65) | 20 (-14, 50) | 37 (3.8, 62) | 13 (-58, 73) |
| S. pneumoniae | cephalosporin | 26 (-6.9, 53) | 25 (-8.1, 52) | 31 (-1.4, 57) | 19 (-14, 48) | 28 (-4.4, 55) | 26 (-6.8, 53) | -13 (-66, 48) |
| Serratia | beta-lactam | 26 (-8.4, 54) | 20 (-15, 50) | 26 (-8.1, 55) | 22 (-12, 51) | 20 (-14, 50) | 23 (-11, 52) | 46 (-7.4, 78) |
| CoNS | beta-lactam | 25 (-5.4, 52) | 30 (-0.96, 55) | 23 (-7.8, 50) | 40 (11, 63) | 21 (-10, 48) | 31 (0.73, 57) | 15 (-36, 59) |
| P. aeruginosa | beta-lactam | 24 (-6.6, 50) | 19 (-11, 46) | 37 (7.8, 60) | 12 (-18, 41) | 25 (-4.8, 51) | 27 (-3.1, 53) | 28 (-21, 66) |
| CoNS | nitrofurantoin | 23 (-9.7, 51) | 11 (-21, 41) | 7.5 (-25, 38) | 23 (-9.1, 51) | 29 (-3.2, 55) | 26 (-7.4, 54) | 16 (-39, 62) |
| E. cloacae | nitrofurantoin | 23 (-10, 51) | 24 (-8.7, 52) | 6.6 (-26, 38) | 22 (-11, 50) | 22 (-11, 50) | 21 (-12, 49) | 27 (-28, 69) |
| E. aerogenes | nitrofurantoin | 22 (-12, 52) | 18 (-17, 49) | 8.5 (-26, 41) | -3.1 (-37, 31) | 21 (-14, 51) | 32 (-2.5, 59) | 65 (18, 88) |
| S. aureus | beta-lactam | 21 (-9.6, 48) | 15 (-16, 43) | 19 (-12, 46) | 16 (-15, 44) | 19 (-12, 46) | 20 (-11, 48) | 48 (3.2, 77) |
| S. pneumoniae | quinolone | 20 (-13, 49) | 19 (-14, 48) | 0.81 (-31, 33) | 25 (-8, 52) | 22 (-10, 51) | 27 (-5.3, 54) | 68 (18, 90) |
| P. mirabilis | quinolone | 20 (-11, 48) | 20 (-11, 47) | 1.5 (-29, 32) | 32 (2.1, 57) | 14 (-17, 42) | 22 (-9, 49) | -14 (-54, 31) |
| P. mirabilis | TMP/SMX | 19 (-12, 47) | 16 (-15, 45) | 19 (-12, 47) | 31 (0.95, 56) | 19 (-12, 47) | 21 (-9.7, 49) | 39 (-5.5, 70) |
| P. aeruginosa | cephalosporin | 18 (-12, 45) | 12 (-18, 40) | 28 (-2.1, 53) | 11 (-20, 39) | 19 (-12, 46) | 22 (-8.9, 49) | 26 (-20, 63) |
| A. baumannii | TMP/SMX | 17 (-16, 47) | 23 (-9.9, 52) | 17 (-16, 47) | 12 (-21, 43) | 22 (-11, 51) | 30 (-3.3, 57) | 13 (-46, 63) |
| CoNS | quinolone | 17 (-16, 47) | 18 (-15, 47) | 28 (-4.4, 56) | 30 (-2.5, 57) | 17 (-16, 47) | 12 (-22, 42) | -0.44 (-50, 49) |
| E. cloacae | TMP/SMX | 16 (-15, 44) | 8.2 (-23, 38) | 14 (-17, 43) | 8.1 (-23, 38) | 16 (-16, 44) | 11 (-20, 40) | 25 (-25, 64) |
| K. pneumoniae | beta-lactam | 15 (-16, 43) | 5.9 (-25, 35) | 15 (-15, 43) | 20 (-11, 47) | 14 (-16, 42) | 12 (-19, 41) | 54 (13, 79) |
| S. aureus | tetracycline | 15 (-16, 44) | -4.6 (-35, 27) | -1.4 (-32, 29) | 29 (-1.8, 55) | 14 (-18, 43) | 13 (-19, 42) | 18 (-31, 60) |
| K. oxytoca | quinolone | 14 (-19, 44) | 16 (-17, 46) | 7.9 (-25, 39) | 31 (-1, 57) | 11 (-21, 42) | 19 (-14, 49) | -23 (-66, 32) |
| E. coli | nitrofurantoin | 13 (-18, 41) | 12 (-19, 41) | -9.9 (-39, 21) | 11 (-20, 40) | 10 (-21, 39) | 9.9 (-22, 39) | -12 (-54, 34) |
| S. pneumoniae | tetracycline | 12 (-21, 43) | -16 (-46, 18) | -4.1 (-36, 29) | -3.4 (-36, 30) | 2.6 (-31, 35) | 12 (-22, 43) | -18 (-76, 55) |
| S. aureus | quinolone | 11 (-20, 40) | 12 (-19, 42) | 9.4 (-22, 39) | 5.4 (-26, 36) | 9.3 (-22, 39) | 6.2 (-25, 37) | -6.9 (-52, 41) |
| Morganella | beta-lactam | 11 (-24, 43) | 3 (-31, 36) | -5.2 (-38, 29) | 0.015 (-34, 34) | 2.5 (-32, 36) | 17 (-18, 48) | 61 (-2.8, 90) |
| C. freundii | TMP/SMX | 10 (-22, 41) | 13 (-19, 43) | 11 (-22, 41) | 39 (8.9, 63) | 5.6 (-26, 37) | 11 (-22, 41) | 32 (-22, 72) |
| P. mirabilis | cephalosporin | 10 (-21, 39) | 9.6 (-21, 39) | 13 (-18, 42) | 4.3 (-26, 34) | 6.9 (-24, 37) | 9.1 (-22, 38) | 22 (-24, 59) |
| E. aerogenes | TMP/SMX | 8.9 (-23, 39) | 11 (-21, 41) | 7.7 (-24, 38) | 10 (-22, 40) | 13 (-19, 43) | 2.9 (-28, 34) | 29 (-22, 68) |
| Serratia | TMP/SMX | 8.3 (-25, 40) | 9.8 (-23, 41) | 9.3 (-24, 40) | 24 (-9.5, 52) | 5.6 (-27, 37) | 2.8 (-30, 35) | 52 (-1.9, 82) |
| K. pneumoniae | nitrofurantoin | 8 (-23, 38) | 1.2 (-30, 32) | -2.7 (-33, 28) | 4.9 (-26, 35) | 6.7 (-25, 37) | 13 (-18, 43) | 8.5 (-40, 53) |
| Serratia | quinolone | 8 (-23, 38) | 9.1 (-22, 39) | -1.3 (-32, 30) | 5.4 (-26, 36) | 4.1 (-27, 34) | 7.7 (-24, 38) | -7.2 (-55, 44) |
| K. pneumoniae | cephalosporin | 7.3 (-23, 36) | 2.4 (-27, 32) | 31 (0.96, 55) | -9 (-38, 21) | 4.6 (-25, 34) | 10 (-20, 39) | -4.3 (-47, 40) |
| S. aureus | cephalosporin | 5.9 (-28, 38) | 1.3 (-32, 34) | 13 (-21, 44) | -5.3 (-38, 29) | 6.5 (-27, 39) | 10 (-25, 43) | 40 (-16, 77) |
| E. faecalis | beta-lactam | 4.3 (-28, 35) | 1.9 (-30, 33) | 9.4 (-23, 40) | -3.1 (-34, 29) | 8.6 (-24, 39) | 3.1 (-29, 34) | -17 (-66, 42) |
| S. aureus | TMP/SMX | 3.7 (-27, 33) | 5.6 (-25, 35) | 4 (-26, 34) | -20 (-47, 11) | 8.2 (-22, 37) | 12 (-19, 41) | -14 (-55, 32) |
| E. faecalis | nitrofurantoin | 3.3 (-30, 36) | -1.4 (-34, 32) | -20 (-50, 14) | 4.2 (-29, 37) | 7.8 (-26, 40) | -10 (-42, 24) | -4.1 (-63, 57) |
| K. oxytoca | TMP/SMX | 3.1 (-29, 35) | -3.1 (-35, 29) | 2.2 (-30, 34) | 15 (-18, 44) | 0.18 (-32, 32) | 2.2 (-31, 35) | 41 (-13, 76) |
| C. freundii | nitrofurantoin | -0.47 (-32, 32) | 0.8 (-31, 33) | -9.2 (-40, 23) | 8.9 (-24, 40) | -1.1 (-33, 31) | -7.3 (-39, 26) | -21 (-65, 34) |
| P. mirabilis | beta-lactam | -1.9 (-32, 29) | -0.69 (-31, 30) | -15 (-44, 16) | 8.2 (-23, 38) | -3.7 (-34, 27) | -2.3 (-32, 28) | -13 (-53, 32) |
| K. oxytoca | beta-lactam | -4.2 (-36, 28) | -13 (-43, 20) | -3 (-35, 29) | 4.5 (-28, 36) | -6.8 (-38, 26) | -4.2 (-37, 29) | 40 (-16, 77) |
| K. pneumoniae | TMP/SMX | -4.6 (-34, 25) | -6.7 (-36, 23) | -5.8 (-35, 24) | -27 (-53, 2.6) | -3.8 (-33, 26) | 6.4 (-24, 36) | 28 (-17, 64) |
| CoNS | tetracycline | -5.5 (-37, 27) | -26 (-53, 6.8) | -22 (-51, 11) | -4.2 (-36, 28) | -19 (-48, 14) | -6.3 (-37, 26) | -2.3 (-53, 49) |
| K. oxytoca | nitrofurantoin | -6.2 (-39, 28) | -17 (-48, 17) | -36 (-62, -2.5) | -7.2 (-40, 27) | -1.7 (-35, 32) | 5.8 (-29, 39) | 0.36 (-51, 51) |
| S. pneumoniae | beta-lactam | -11 (-41, 22) | -7.5 (-38, 25) | -1.9 (-33, 30) | -3.8 (-35, 28) | -6.7 (-37, 25) | -3.3 (-34, 29) | 35 (-28, 77) |

TMP/SMX: trimethoprim/sulfamethoxazole.

CoNS: coagulase-negative *Staphylococcus*.

**Table 3: Systematic differences in use-resistance correlations.** The first column shows a pair of approaches that were compared in a sensitivity analysis. The second column shows the correlation between the values (i.e., the Spearman’s ρ between use and resistance) predicted by the analysis using the two approaches mentioned in the comparison. Children only: MarketScan data including only members 15 and younger. HRR: hospital referral region. IP: inpatient. OP/ER: outpatient/emergency room.

| **Comparison** | **Correlation between values (Pearson’s *r*, %)** | **Median difference in absolute value of correlation coefficient (percentage points, 95% CI, Wilcoxon test)** |
| --- | --- | --- |
| Prescription fills (main data) vs. days supply | 95 | –1.9 (–3.1, –0.72) |
| Adults and children (main data) vs. children only | 85 | –0.70 (–3.0, 1.6) |
| MarketScan (main data) vs. Medicare | 84 | –1.5 (–3.9, 1.4) |
| Medicare state-level vs. HRR-level | 79 | –6.4 (–9.4, –3.3) |
| All resistance data vs. not IP isolates only | 97 | 1.1 (0.011, 2.2) |
| All resistance data vs. OP/ER isolates only | 36 | 3.9 (–1.9, 9.9) |

**Table 4: Correlations between first use and repeat use.** Variance inflation factors greater than 5 or 10 are sometimes used as a rule of thumb for determining when predictors in a multiple regression are problematically collinear.

| **Drug group** | **Correlation between first and repeat use (Pearson’s *r*, %)** | **Variance inflation factor** |
| --- | --- | --- |
| cephalosporin | 97.5 | 19.9 |
| quinolone | 96.7 | 15.5 |
| macrolide | 96.5 | 14.6 |
| β-lactam | 94.4 | 9.2 |
| nitrofurantoin | 88.4 | 4.6 |
| TMP/SMX | 87.4 | 4.2 |
| tetracycline | 44.4 | 1.2 |

**Table 5: Sensitivity of multiple regression coefficients.** Children only: MarketScan data including only members 15 and younger. HRR: hospital referral region. IP: inpatient. OP/ER: outpatient/emergency room.

| **Data source/population** | **Proportion of first use regression coefficients that are positive (%, 95% CI)** | **Proportion of repeat use regression coefficients that are negative (%, 95% CI)** |
| --- | --- | --- |
| MarketScan (main data) | 75 (63, 84) | 61 (49, 72) |
| Children only | 74 (62, 83) | 68 (56, 79) |
| Days supply | 74 (62, 83) | 69 (57, 80) |
| Medicare state-level | 81 (70, 89) | 67 (55, 77) |
| Medicare HRR-level | 78 (66, 87) | 68 (56, 79) |
| Not IP isolates only | 78 (66, 87) | 62 (50, 74) |
| OP/ER isolates only | 67 (55, 77) | 56 (43, 67) |

**Table 6: Generic antibiotic formulations.** Antibiotic claims were identified in the MarketScan data using these generic names.

| **Generic name** | **Antibiotic** |
| --- | --- |
| Alatrofloxacin Mesylate | alatrofloxacin |
| Amdinocillin | amdinocillin |
| Amikacin Sulfate | amikacin |
| Amoxicillin | amoxicillin |
| Amoxicillin;Clarithromycin;Lansoprazole | amoxicillin/clarithromycin |
| Amoxicillin/Clavulanate Potassium | amoxicillin/clavulanate |
| Ampicillin | ampicillin |
| Ampicillin Sodium | ampicillin |
| Ampicillin Sodium/Sulbactam Sodium | ampicillin/sulbactam |
| Ampicillin/Probenecid | ampicillin |
| Azithromycin | azithromycin |
| Azithromycin Dihydrate | azithromycin |
| Azithromycin/Dextrose | azithromycin |
| Azlocillin Sodium | azlocillin |
| Azo-Methenamine Mandelate | methenamine |
| Azo-Sulfamethoxazole | sulfamethoxazole |
| Aztreonam | aztreonam |
| Bacampicillin Hydrochloride | bacampicillin |
| Bacitracin | bacitracin |
| Bedaquiline Fumarate | bedaquiline |
| Benzoic Acid/Hyoscyamine Sulf/Methenamine/Methylen | methenamine |
| Benzoic Acid/Methenamine/Sodium Salicylate | methenamine |
| Bi Subcitrate K/Metronidazole/Tetracycline HCl | metronidazole/tetracycline |
| Bi Subsalicylate;Metronidazole;Tetracycline HCl | metronidazole/tetracycline |
| Bi Subsalicylate/Metronidazole/Tetracycline HCl | metronidazole/tetracycline |
| Capreomycin | capreomycin |
| Carbenicillin Disodium | carbenicillin |
| Carbenicillin Indanyl Sodium | carbenicillin |
| Cefaclor | cefaclor |
| Cefadroxil | cefadroxil |
| Cefamandole Nafate | cefamandole |
| Cefazolin | cefazolin |
| Cefazolin Sodium | cefazolin |
| Cefazolin Sodium/Dextrose | cefazolin |
| Cefazolin Sodium/Sodium Chloride | cefazolin |
| Cefdinir | cefdinir |
| Cefditoren Pivoxil | cefditoren |
| Cefepime Hydrochloride | cefepime |
| Cefixime | cefixime |
| Cefmetazole Sodium | cefmetazole |
| Cefonicid Sodium | cefonicid |
| Cefoperazone Sodium | cefoperazone |
| Ceforanide | ceforanide |
| Cefotaxime Sodium | cefotaxime |
| Cefotetan Disodium | cefotetan |
| Cefoxitin | cefoxitin |
| Cefoxitin Sodium | cefoxitin |
| Cefpodoxime Proxetil | cefpodoxime |
| Cefprozil | cefprozil |
| Ceftaroline Fosamil | ceftaroline |
| Ceftazidime | ceftazidime |
| Ceftazidime Sodium/Dextrose | ceftazidime |
| Ceftibuten | ceftibuten |
| Ceftizoxime Sodium | ceftizoxime |
| Ceftriaxone Sodium | ceftriaxone |
| Ceftriaxone Sodium/Lidocaine | ceftriaxone |
| Cefuroxime Axetil | cefuroxime |
| Cefuroxime Sodium | cefuroxime |
| Cephalexin | cephalexin |
| Cephalexin Hydrochloride | cephalexin |
| Cephalothin Sodium | cephalothin |
| Cephapirin Sodium | cephapirin |
| Cephradine | cephradine |
| Chloramphenicol | chloramphenicol |
| Chloramphenicol Palmitate | chloramphenicol |
| Chloramphenicol Sodium Succinate | chloramphenicol |
| Cilastatin Sodium/Imipenem | imipenem |
| Cinoxacin | cinoxacin |
| Ciprofloxacin | ciprofloxacin |
| Ciprofloxacin Hydrochloride | ciprofloxacin |
| Ciprofloxacin/Ciprofloxacin Hydrochloride | ciprofloxacin |
| Clarithromycin | clarithromycin |
| Clavulanate Potassium/Ticarcillin Disodium | ticarcillin/clavulanate |
| Clindamycin Hydrochloride | clindamycin |
| Clindamycin Palmitate Hydrochloride | clindamycin |
| Clindamycin Phosphate | clindamycin |
| Clindamycin Phosphate;Cleanser and Moisturizer | clindamycin |
| Clindamycin Phosphate/Dextrose | clindamycin |
| Clofazimine | clofazimine |
| Cloxacillin Sodium | cloxacillin |
| Colistimethate Sodium | colistin |
| Colistin Sulfate | colistin |
| Cyclacillin | cyclacillin |
| Cycloserine | cycloserine |
| Dalfopristin/Quinupristin | dalfopristin/quinupristin |
| Dapsone | dapsone |
| Daptomycin | daptomycin |
| Demeclocycline Hydrochloride | demeclocycline |
| Dextrose/Levofloxacin | levofloxacin |
| Dextrose/Vancomycin Hydrochloride | vancomycin |
| Dicloxacillin Sodium | dicloxacillin |
| Dirithromycin | dirithromycin |
| Doripenem | doripenem |
| Doxycycline | doxycycline |
| Doxycycline Calcium | doxycycline |
| Doxycycline Hyclate | doxycycline |
| Doxycycline Hyclate;Eyelid Cleanser;Spray, Multi I | doxycycline |
| Doxycycline;Flaxseed Oil/Omega-3 Fatty Acids/Vit E | doxycycline |
| Doxycycline;Octinoxate/Zinc Oxide;Salicylic Acid | doxycycline |
| Enoxacin | enoxacin |
| Ertapenem Sodium | ertapenem |
| Erythromycin | erythromycin |
| Erythromycin Estolate | erythromycin |
| Erythromycin Ethylsuccinate | erythromycin |
| Erythromycin Ethylsuccinate/Sulfisoxazole Acetyl | erythromycin/sulfisoxazole |
| Erythromycin Gluceptate | erythromycin |
| Erythromycin Lactobionate | erythromycin |
| Erythromycin Stearate | erythromycin |
| Ethambutol Hydrochloride | ethambutol |
| Ethionamide | ethionamide |
| Fidaxomicin | fidaxomicin |
| Fosfomycin Tromethamine | fosfomycin |
| Furazolidone | furazolidone |
| Gatifloxacin | gatifloxacin |
| Gemifloxacin Mesylate | gemifloxacin |
| Gentamicin Sulfate | gentamicin |
| Gentamicin Sulfate/Sodium Chloride | gentamicin |
| Grepafloxacin Hydrochloride | grepafloxacin |
| Hetacillin | hetacillin |
| Hyoscyamine/Methenamine Mandelate | methenamine |
| Isoniazid | isoniazid |
| Isoniazid W/Pyridoxine | isoniazid |
| Isoniazid/Pyrazinamide/Rifampin | isoniazid/pyrazinamide/rifampin |
| Isoniazid/Rifampin | isoniazid/rifampin |
| Kanamycin Sulfate | kanamycin |
| Levofloxacin | levofloxacin |
| Lincomycin Hydrochloride | lincomycin |
| Linezolid | linezolid |
| Lomefloxacin Hydrochloride | lomefloxacin |
| Loracarbef | loracarbef |
| Meropenem | meropenem |
| Methacycline Hydrocholride | methacycline |
| Methenamine Combinations | methenamine |
| Methenamine Hippurate | methenamine |
| Methenamine Mandelate | methenamine |
| Methenamine Mandelate/Potassium Phosphate | methenamine |
| Methenamine Mandelate/Sodium Phosphate, Monobasic | methenamine |
| Methenamine/Sodium Phosphate, Monobasic | methenamine |
| Methenamine/Sodium Salicylate | methenamine |
| Methicillin Sodium | methicillin |
| Metronidazole | metronidazole |
| Metronidazole Hydrochloride | metronidazole |
| Mezlocillin Sodium | mezlocillin |
| Minocycline Hydrochloride | minocycline |
| Moxalactam | moxalactam |
| Moxifloxacin Hydrochloride | moxifloxacin |
| Nafcillin Sodium | nafcillin |
| Nalidixic Acid | nalidixic acid |
| Neomycin Sulfate | neomycin |
| Neomycin Sulfate/Polymyxin B Sulfate | neomycin/polymyxin b |
| Nitrofurantoin | nitrofurantoin |
| Nitrofurantoin Monohydrate/Nitrofurantoin, Macro | nitrofurantoin |
| Nitrofurantoin, Macrocrystals | nitrofurantoin |
| Norfloxacin | norfloxacin |
| Ofloxacin | ofloxacin |
| Oxacillin Sodium | oxacillin |
| Oxytetracycline Hydrochloride | oxytetracycline |
| Paromomycin Sulfate | paromomycin |
| Pen. G. Proc/Probenecid | penicillin g |
| Penicillin G Benzathine | penicillin g |
| Penicillin G Benzathine/Penicillin G Procaine | penicillin g |
| Penicillin G Potassium | penicillin g |
| Penicillin G Potassium/Sodium Chloride | penicillin g |
| Penicillin G Procaine | penicillin g |
| Penicillin G Sodium | penicillin g |
| Penicillin V Potassium | penicillin v |
| Phenazopyridine Hydrochloride/Sulfamethoxazole | sulfamethoxazole |
| Phenazopyridine Hydrochloride/Sulfisoxazole | sulfisoxazole |
| Phenazopyridine/Sulfamethizole | sulfamethizole |
| Piperacillin Sodium | piperacillin |
| Piperacillin Sodium/Tazobactam Sodium | piperacillin/tazobactam |
| Polymyxin B Sulfate | polymyxin b |
| Pyrazinamide | pyrazinamide |
| Retapamulin | retapamulin |
| Rifabutin | rifabutin |
| Rifampin | rifampin |
| Rifapentine | rifapentine |
| Rifaximin | rifaximin |
| Sodium Chloride/Tobramycin Sulfate | tobramycin |
| Sodium Chloride/Vancomycin Hydrochloride | vancomycin |
| Sparfloxacin | sparfloxacin |
| Spectinomycin Hydrochloride | spectinomycin |
| Streptomycin Sulfate | streptomycin |
| Sulfacytine | sulfacytine |
| Sulfadiazine | sulfadiazine |
| Sulfadiazine/Sulfamerazine/Sulfamethazine | sulfadiazine/sulfamerazine/sulfamethazine |
| Sulfamethizole | sulfamethizole |
| Sulfamethoxazole | sulfamethoxazole |
| Sulfamethoxazole/Trimethoprim | trimethroprim/sulfamethoxazole |
| Sulfasalazine | sulfasalazine |
| Sulfisoxazole | sulfisoxazole |
| Sulfisoxazole Acetyl | sulfisoxazole |
| Tedizolid Phosphate | tedizolid |
| Telavancin Hydrochloride | telavancin |
| Telithromycin | telithromycin |
| Temafloxacin Hcl | temafloxacin |
| Tetracycline Hydrochloride | tetracycline |
| Tetracycline/Amphotericin | tetracycline |
| Ticarcillin Disodium | ticarcillin |
| Tigecycline | tigecycline |
| Tobramycin | tobramycin |
| Tobramycin Sulfate | tobramycin |
| Trimethoprim | trimethoprim |
| Trimethoprim Hydrochloride | trimethoprim |
| Troleandomycin | troleandomycin |
| Trovafloxacin Mesylate | trovafloxacin |
| Vancomycin Hydrochloride | vancomycin |

**Table 7: Oral and injected drug forms.** MarketScan antibiotic claims were filtered for these forms.

| **Drug form** |
| --- |
| Capsule |
| Capsule, Delayed Release |
| Capsule, Extended Release |
| Injectable |
| Powder |
| Powder for Injection |
| Powder for Solution |
| Powder for Suspension |
| Powder for Suspension, Extende |
| Solution |
| Suspension |
| Tablet |
| Tablet for Suspension |
| Tablet, Chewable |
| Tablet, Delayed Release |
| Tablet, Enteric Coated |
| Tablet, Extended Release |
